# Self-Control, Consideration of Future Consequences, and Internet Addiction among Chinese Adolescents: The Moderating Effect of Deviant Peer Affiliation

**DOI:** 10.3390/ijerph18179026

**Published:** 2021-08-27

**Authors:** Jingjing Li, Yanhan Chen, Jiachen Lu, Weidong Li, Chengfu Yu

**Affiliations:** 1 School of Psychology, South China Normal University, Guangzhou 510631, China; 2020010222@m.scnu.edu.cn (J.L.); 2019022970@m.scnu.edu.cn (Y.C.); 2 School of Physical Education, Guangzhou University, Guangzhou 510006, China; dongminjing@126.com; 3 Research Center of Adolescent Psychology and Behavior, Department of Psychology, School of Education, Guangzhou University, Guangzhou 510006, China; yuchengfu@gzhu.edu.cn

**Keywords:** self-control, internet addiction, consideration of future consequences, deviant peer affiliation

## Abstract

Although a large number of studies have indicated that self-control was an important predictive factor for adolescent internet addiction, the moderating and mediating mechanisms underlying this relationship remain unclear. To address this research gap, the present study, according to social learning theory and the organism–environment interaction theory, tested whether consideration of future consequences mediated the relation between self-control and adolescent internet addiction and whether deviant peer affiliation moderated the relationship between consideration of future consequences and internet addiction. Using longitudinal tracking (two-time points and the interval is 6 months). Three middle schools in Guangzhou were randomly selected. The participants were 1182 students ranging in age from 12 to 15 years (average age: 14.16, SD = 1.29) from three middle schools in Guangzhou (651 boys and 531 girls) in Guangdong Province. Results showed that consideration of future consequences mediated the relationship between self-control and internet addiction. Furthermore, the relationship between future consequence consideration and internet addiction was moderated by deviant peer affiliation. These findings highlighted the potential mediating role of consideration of future consequences in linking self-control to adolescent internet addiction. We also found high deviant peer affiliation weakens the protective effect of future consequence consideration on Internet addiction. This study may provide support for adolescent Internet addiction prevention and have some educational implications.

## 1. Introduction

The development of networked information technology and the rise of the information society has led to the ubiquity of the internet, transforming nearly every aspect of human lives. For teenagers, computers have become a necessary communication tool. Through rich digital language and massive information resources, the internet breaks through the inherent space and time boundary, opening up an infinite and vast communication space and offering various forms of opportunities for people [1,2]. However, the internet is like a double-edged sword in that it brings convenience and abundant resources for adolescences’ study and life but also carries a certain negative impact on the development of their thoughts and behaviors. For example, overuse of the internet leads to internet addiction, defined as excessive and uncontrolled internet use [3]. Internet addiction not only seriously affects adolescents’ normal study and life habits but also damages their physical and mental health development [4,5]. Internet addiction in adolescents has been attributed to lead to absenteeism, declining academic performance, depression, and even extreme ideas and behaviors, such as suicide and crime [6,7]. Therefore, practical remedial and prevention programs must be established, with consideration for the scientific identification of the protective and risk factors and underlying mechanisms for adolescent internet addiction.

### 1.1. Self-Control and Adolescent Internet Addiction

The ability of human self-control can be regarded as one of the most powerful and beneficial adaptations in human psychology. Individuals are the happiest and healthiest when they and the environment are in the best fit, and this fit can be significantly improved by changing themselves to adapt to the external environment. [8,9]. It plays a crucial role in bringing happiness, success, and life satisfaction. Moreover, self-control enables individuals to establish long-term plans and develop a rational way of thinking [9,10]. Individuals with high self-control typically have a clear plan for their future development, better academic performance, rational thinking, and strong will [9,11]. Meanwhile, low self-control is closely related to substance abuse, addictive behavior, and maladjustment [12].

Empirical researches have indicated that self-control is significantly associated with internet addiction. People with high self-control are unlikely to become addicted to the internet, whereas those with low self-control have an increased likelihood of developing internet addiction [12,13,14]. A reason is that internet addiction can be regarded as a failure of self-control. In studies with a self-depletion condition (the lack of self-control), individuals in this group show more impulse and irrational decision-making [15,16]. Internet addiction is also an impulse-control disorder [17,18]. According to social learning theory [19], an individual’s self-regulatory system determines their behavior and is related to internet addiction. Self-control enables people to be aware of their behavioral consequences; therefore, the lack of self-control is one of the most important risk factors for internet addiction [12,13,14]. Previous studies have also confirmed that internet addiction is positively correlated with low self-control [12,20].

### 1.2. Consideration of Future Consequences as Potential Mediator

Consideration of future consequences, referring to the degree to which a person deliberates on future outcomes before engaging in a behavior [21], therefore, individuals can adjust their current behaviors to achieve desired goals by considering future consequences. It has been related to serious problematic behaviors, including risky behavior and substance abuse [22,23]. We speculated that consideration of future consequences could mediate the relationship between self-control and adolescent internet addiction, based on the following.

First, self-control influences people’s consideration of future consequences. According to self-control theory [24], an important characteristic of self-control is delayed gratification. Individuals with low self-control have difficulty resisting immediate temptation, tend to be satisfied in the present, and lack consideration for long-term goals and future consequences [25]. In contrast, high self-control individuals are more focused on future consequences and can resist current temptations to achieve long-term goals [26]. Self-control theory also states that low self-control is found in unprepared individuals with less focus on long-term pursuits [24]. Indeed, high self-control individuals are more likely to show higher levels of consideration for future consequences compared with their low self-control counterparts [27]. Second, individuals’ attitudes toward future outcomes affect their behavior. A high level of consideration for future consequences is prone to guide adolescents’ behavior with long-term goals; these adolescents are unlikely to be influenced by immediate pleasure (such as being addicted to the internet). However, adolescents with a low level of consideration of future consequences typically focus more on immediate rewards and think less about the future consequences of present behavior [27,28]. Avoiding internet addiction would thus require individuals to suppress their current pleasures to promote their long-term development. Therefore, compared with adolescents with low future consideration, adolescence with high future outcome considerations tend to engage in activities that they believe will lead to good outcomes in the future. Based on this, we put forward the following hypothesis:

**Hypothesis** **1.** *Consideration of future consequences will mediate the relationship between self-control and adolescent internet addiction.*

### 1.3. Deviant Peer Affiliation as Potential Moderator

Although consideration of future consequences is generally believed to be a protective factor for avoiding internet addiction [12,20], it might not have a positive influence on all adolescents. According to individual–environment interaction model [29], adolescent internet addiction is influenced by the interaction effect between individual (e.g., consideration of future consequences) and environmental factors (e.g., deviant peer affiliation), and individuals differ in their behavior depending on their environment. Thus, deviant peer affiliation, as an important environmental factor, may play a moderating role between individual variables and behavior.

Deviant peer affiliation means making friends with peers who break laws and social norms [30]. Deviant peer affiliation is one of the most crucial predictors for adolescent problem behaviors, such as substance addiction and aggression [31]. Recent researches have shown that deviant peer affiliation was also an indicator for predicting internet addiction among adolescents [32]. According to social learning theory [33], affiliating with deviant peers, the characteristics of deviant peer groups will affect individuals, and problematic behavior will also occur through imitating and observing their peers’ problem behavior (e.g., problematic internet use; alcohol abuse; drug use; smoking, etc.). Meanwhile, low consideration of future consequences is also the basis for many problematic behaviors. Low future consequence consideration individuals are more likely to engage in aggression, impulsivity, delinquency [34,35]. Based on the risk-enhancing model [36], when two risk factors coexist, their negative effects will be mutually reinforcing. This may be indicated that affiliating with deviant peers makes adolescence learn delinquent behaviors from their peers, thus reducing the thinking of their behavior consequences. And it makes them difficult to analyze problems rationally, and effectively deal with conflicts such as impulsiveness and risk-taking caused by external stimuli. Previous studies showed that deviant peer affiliation interacts with future consequence consideration. The more adolescents affiliate with deviant peers, the more they imitate and learn from peer problem behaviors, pursue immediate stimulation and enjoyment, and the less they consider the consequences of behaviors [37]. This indicated that a high level of deviant peer affiliation may weaken the protective effect of future consequence considerations on internet addiction, while a low level of deviant peer affiliation may buffer the protective effect. Based on these, we put forward the following hypothesis:

**Hypothesis** **2.** *Deviant peer affiliation will moderate the link between consideration of future consequences and adolescent internet addiction.*

**Figure 1:**
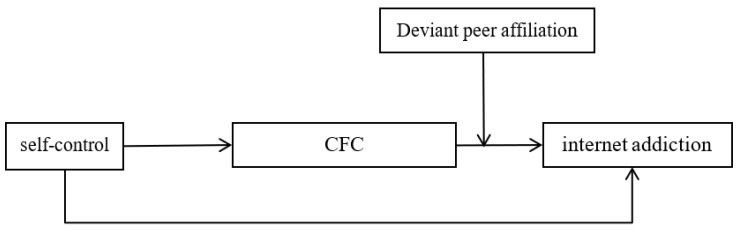
The proposed mediated moderation model. Note: CFC = Consideration of Future Consequences.

In summary, the current study integrated two theories (social learning theory and organism–environment interaction theory) and a longitudinal trace of two-time points (T1 and T2), the interval is half a year to account for the mechanisms of how and when self-control is linked to adolescent internet addiction. The proposed mediated moderation model see Figure 1.

## 2. Methods

### 2.1. Procedure

In this study, the same group of students was tested using a questionnaire in July 2019 (T1) and January 2020 (T2) in a longitudinal investigation, and the interval is 6 months. At T1, adolescents were asked to complete the Brief Self-Control Scale (BSCS), Consideration of Future Consequences Scale (CFC), and a demographic questionnaire. At T2, the same adolescents were asked to complete the Deviant Peer Affiliations Scale (DPA) and Young’s Diagnostic Questionnaire (YDQ).

Three public junior middle schools were randomly selected from Guangzhou to conduct a questionnaire survey. The test was conducted collectively in a class, with undergraduates and postgraduates majoring in psychology as the test subjects. First of all, before the questionnaire is distributed, the students gather in a quiet classroom, and the examiners read out the questionnaire instruction to the participants, and the participants signed an informed consent form. During the process of filling out the questionnaire, teachers were on the spot to maintain discipline and answer questions from the participants. After participants finished the questionnaire, the questionnaire was recovered by the examiners, and the questionnaire was checked on the spot and invalid questionnaires were eliminated. A total of 1200 questionnaires were returned, of which 1182 were valid questionnaires, with a recovery rate of 98.5%.

### 2.2. Participants

This study was approved by the Ethics Committee of the School of Education at Guangzhou University (No. GZHU2019017). Students were from three different middle schools in Guangzhou, Guangdong province. The participants were 1182 students ranging in age from 12 to 15 years (average age: 14.16, SD = 1.29) from three middle schools in Guangzhou (651 boys and 531 girls) in Guangdong Province. Among all the participants, 31.1% self-reported that they lived in cities, 16.6% of those whose fathers had a bachelor’s degree or above, and 12.8% of their mothers had a bachelor’s degree or above.

### 2.3. Brief Self-Control Scale

The BSCS, developed by Tangney et al. (2004) [38], is widely used for measuring the level of individual self-control. Higher scores indicate a higher level of individual self-control, 13 items on this scale and has been proven to be reliable and valid in Chinese teenagers [11]. It includes items such as “I’m good at resisting temptation” and “I’m lazy.” The Cronbach’s α coefficient in this study was 0.86.

### 2.4. Consideration of Future Consequences Scale

This scale, developed by Strathman, Gleicher, Boninger, and Edwards (1994) [21] and revised by Dou and Wang (2016), contains 12 questions under the two dimensions of consideration of immediate (7 items) and future factors (5 items). Each item is scored on a seven-point scale, from 1 = *very unlike me* to 7 = *very like me*. Higher scores indicate more thought given to future outcomes. An example item is “I am willing to sacrifice my present happiness and joy for the sake of my future success.” This scale has been proven to be reliable and valid in Chinese participants [27]. In our study, Cronbach’s α coefficient was 0.89.

### 2.5. Deviant Peer Affiliations

From the Peer Group Characteristics Questionnaire compiled by Elliot et al. (1985) [39], and this scale has been proven to be reliable and valid in Chinese participants [5]. we used 10 items to measure the number of peers who showed various problem behaviors in the circle of the participants using a three-point score: 1 = *no one*, 2 = *one person*, and 3 = *two or more people*. In the present research, Cronbach’s α coefficient is 0.84.

### 2.6. Young’s Diagnostic Questionnaire

This scale, developed by Young et al. (2004) [40], is widely used to measure the level of Internet addiction among adolescents and has been proven to be reliable and valid in Chinese teenagers [7]. Items are scored on a three-point scale. Those who meet five of the items can be diagnosed as having internet addiction. The YDQ has good reliability and validity and is suitable for teenagers and college students. An example item is “Are you addicted to the internet?" In the present research, Cronbach’s α coefficient is 0.78.

### 2.7. Statistical Analyses

SPSS 21.0 was used for the descriptive statistical analysis. Moreover, mediation and moderation effects were tested with SPSS PROCESS V3.4 macro developed by Hayes (2013). Self-control, consideration of future consequences, deviant peer affiliation, and internet addiction have entered the model as an independent variable, mediator, moderator, and dependent variable, respectively. Effects obtained from the total effect model and the indirect effect were reported. Bootstrapping (N = 1000) was employed and the 95% confidence interval (CI) was used to justify the significance of the moderating mediating effect. If the 95% CI does not include 0, then a significant mediation effect is tenable.

## 3. Results

### 3.1. Preliminary Analyses

Results in Table 1 illustrated that BSCS is positively associated with CFC, and BSCS is negatively associated with YDQ and DPA. CFC was negatively correlated with YDQ and DPA. Moreover, YDQ was positively correlated with DPA.

### 3.2. Testing for the Moderated Mediation

**Figure 2:**
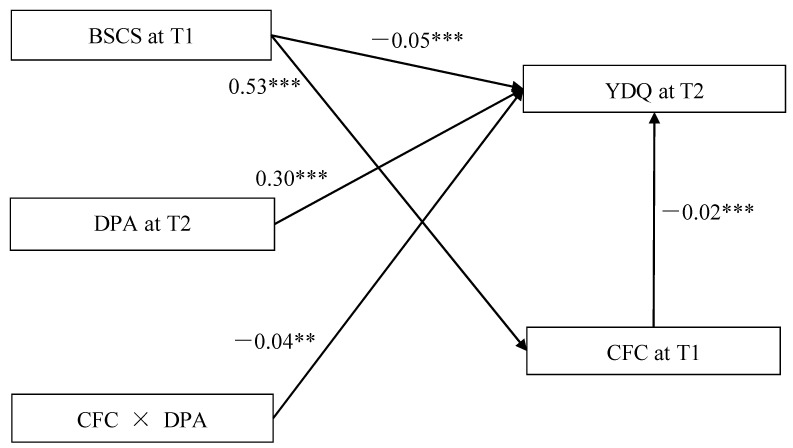
Model of the moderating role of DPA in the indirect relation between BSCS and YDQ. Note: ** *p* < 0.01, *** *p* < 0.001. BSCS = Brief Self-Control Scale, CFC = Consideration of Future Consequences Scale, DPA = Deviant Peer Affiliations Scale, YDQ = Young’s Diagnostic Questionnaire. Covariates including sex, home residence, and the education level of parents.

**Figure 3:**
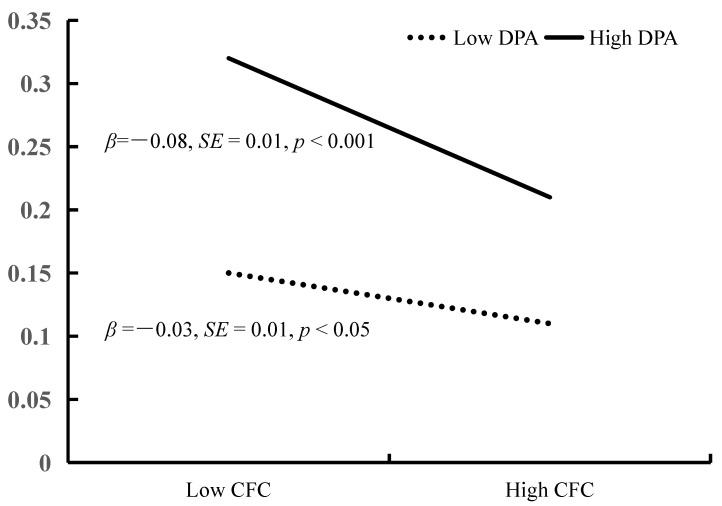
YDQ among adolescents as a function of CFC and DPA. Note: BSCS = Brief Self-Control Scale, CFC = Consideration of Future Consequences Scale, DPA = Deviant Peer Affiliations Scale, YDQ = Young’s Diagnostic Questionnaire.

The model 14 of PROCESS 3.4 macro(Hayes, 2013) is used to test the moderated mediation model. The Moderated Mediation testing is showed in Figure 2. After controlling for sex, home residence and education level of parents, the bias-corrected percentile bootstrap results indicated that the indirect effect of BSCS on YDQ through CFC was moderated by DPA. Specifically, DPA moderated the association between CFC and YDQ (*β* = −0.04, *t* = −3.06, *p* < 0.01, 95% CI = [−0.07, −0.02]). We conducted a simple slopes test, and as depicted in Figure 3, the negative association between CFC and YDQ was significantly stronger among adolescents with higher DPA (1 *SD* above the mean; *β* = −0.06, *t* = −3.91, *p* < 0.001, 95% CI = [−0.09, −0.03]) than among adolescents with lower DPA (1 *SD* below the mean; *β* = −0.05, *t* = −3.25, *p* < 0.05, 95% CI = [−0.08, −0.02]). BSCS showed a significant positive association with CFC (*β* = 0.53, *t* = 17.43, *p* <0.001, 95% CI = [0.47, 0.59]), and DPA had a significant positive relation with YDQ (*β* = 0.30, *t* = 4.64, *p* < 0.001, 95% CI = [0.17, 0.42]).

Finally, the bias-corrected percentile bootstrap results indicated that the indirect link between BSCS and YDQ via CFC was stronger among adolescents with high DPA (indirect effect = −0.03, SE = 0.01, 95% CI = [−0.05, −0.01]) than among those with low DPA (indirect effect = −0.01, SE = 0.01, 95% CI = [−0.03, 0.01]). Therefore, the mediating effect of CFC between BSCS and YDQ was moderated by DPA.

## 4. Discussion

This study examined how self-control is related to internet addiction and whether the association varies according to adolescents’ deviant peer affiliation. We found that adolescents with high self-control showed a high level of consideration of future outcomes, which in turn reduced internet addiction. In addition, this indirect link was moderated by deviant peer affiliations. In line with hypothesis 1, this result showed that consideration of future consequences mediated the relation between self-control and internet addiction in adolescents. According to self-control theory [24], high self-control adolescence are more likely to resist current temptations, make their behavior subordinate to long-term goals, and thus think more about the future consequences of their behavior. Individuals with high levels of consideration of future consequences are more focused on long-term outcomes regardless of the short-term sacrifice, and thus have a low likelihood of being addicted to the internet. Prior studies support this path and have shown a strong association between future orientation and internet addiction [41].

Research has explored the influence of self-control or consideration of future consequences on adolescent internet addiction [12,13]. To expand the current understanding of these associations, the present study examined these indicators simultaneously. We found that consideration of future consequences was a significant explanatory mechanism for the reason that self-control could reduce internet addiction among adolescents. Indeed, consideration of future consequences is an important psychosocial asset that can be cultivated via high self-control. This finding has a significant contribution to adolescent internet addiction prevention.

Our results supported hypothesis 2 and were consistent with organism–environment interaction theory [29]: internet addiction was influenced by the interaction effect between individual and environmental factors. Deviant peer affiliations could significantly enhance the adverse effects of low consideration of future consequences on adolescent internet addiction. First, for adolescents with low deviant peer affiliation, internet addiction tendency was lower than that for those with high deviant peer affiliation. Second, the internet addiction tendency of adolescents with high deviant peer affiliation decreased with an increase in consideration of future consequences. This finding may be because adolescents affiliated with deviant peers tend to be more interested in immediate pleasures than in considering the future consequences of their immediate pleasures. In contrast, adolescents with low deviant peer affiliation have good self-control, which, to some extent, can relieve the adverse influence of low consideration of future consequences [42,43,44]. Therefore, the contribution of consideration of future consequences and deviant peer affiliation to internet addiction should not be viewed independently but considered together. An implication is that researchers should thus address the different factors influencing internet addiction simultaneously.

Our study also highlighted the importance of moderated mediation model. Compared with a simple mediating model, our model provided specific information regarding mediating effects that might not apply to every adolescent, thereby offering better guidance for an internet addiction intervention. At T1, we measured the moderating effect of deviant peer affiliation, which was found to be non-significant, indicating that the moderating role of deviant peer affiliation may be immediate.

The present study has the following educational implications. First of all, for individuals, the quality of peer affiliation has an important impact on the shaping of individual thought and behavior. Affiliating with high-quality peers is beneficial to individuals’ social adaptation and physical and mental health development. On the contrary, deviant peer affiliating make adolescence more likely to learn the problematic behavior from their peers. Therefore, it is significant for adolescence to choose their peers consciously and integrate into a positive and harmonious group of peers. Secondly, the contact between adolescents and their peers becomes more and more close during adolescence, and the impact of peers cannot be ignored. While giving children freedom and space to make friends, parents should also pay attention to the quality of children’s interpersonal interaction. Through encouragement and supportive suggestions, parents should encourage teenagers to choose high-quality friends and participant in positive and beneficial social activities. In addition, teachers should pay attention to the cultivation of peer environment, organize more collective activities that are conducive to the interaction of student groups, so that each student can integrate into peer groups and school life. Finally, teachers should pay attention to students’ situations in real-time, cultivating and improving students’ self-regulation ability, and intervention to students in need, and pay attention to their interpersonal communication and physical and mental health development.

Our study had some limitations. First, longitudinal tracking was adopted in this study, which, to some extent, avoided the shortcomings of cross-sectional studies. However, only two-time points were measured; thus, the stable tendency of self-control and internet addiction could not be captured. Future studies can use additional time points to explore further the relation between these two factors. Second, this study only collected data through self-report methods; future research could use multi-agent assessment methods to collect relevant data reported by parents, peers, and teachers. Finally, the possible moderating mechanism between self-control and adolescent internet addiction should be investigated. For example, sensation seeking is associated with substance and non-substance addiction. The meta-analysis found that sensation seeking was associated with internet addiction, and that sensation seeking in adolescence predicted substance addiction in adulthood. Future research can consider sensation-seeking [45]. Given the complex interactions among different ecosystems, family is also an important area of individual life and plays an important role in adolescent psychological and behavioral development, family-related factors (effective parental behavior control; family emotional warmth; family atmosphere, etc.) are worth exploring.

## 5. Conclusions

By examining a moderated mediation model that accounted for both environmental (deviant peer affiliation) and individual (self-control) factors simultaneously, the current study elucidated when and how high self-control would reduce internet addiction in adolescents. Overall, high self-control can promote adolescents’ consideration of future consequences, which in turn can reduce internet addiction. Moreover, the conducive effect of consideration of future consequences may be weakened by a high level of deviant peer affiliation.

## Figures and Tables

**Table 1:** Descriptive statistics and correlations for all variables.

Variables	1	2	3	4
1. BSCS	1.00			
2. CFC	0.46 **	1.00		
3. YDQ	−0.31 **	−0.20 **	1.00	
4. DPA	−0.23 **	−0.11 **	0.33 **	1.00
*Mean*	3.13	4.40	0.20	1.42
*SD*	0.56	0.65	0.23	0.56

*Note*: ** *p* < 0.01. BSCS = Brief Self-Control Scale, CFC = Consideration of Future Consequences Scale, DPA = Deviant Peer Affiliations Scale, YDQ = Young’s Diagnostic Questionnaire.

## Data Availability

The data presented in this study are available on request from the corresponding author.
